# Germinal Centre Shutdown

**DOI:** 10.3389/fimmu.2021.705240

**Published:** 2021-07-07

**Authors:** Theinmozhi Arulraj, Sebastian C. Binder, Philippe A. Robert, Michael Meyer-Hermann

**Affiliations:** ^1^ Department of Systems Immunology, Braunschweig Integrated Centre of Systems Biology, Helmholtz Centre for Infection Research, Braunschweig, Germany; ^2^ Department of Immunology, University of Oslo, Oslo, Norway; ^3^ Institute for Biochemistry, Biotechnology and Bioinformatics, Technische Universität Braunschweig, Braunschweig, Germany

**Keywords:** germinal centre shutdown, vaccination, chronic germinal centres, B cell lymphoma, ectopic germinal centres, antibody responses

## Abstract

Germinal Centres (GCs) are transient structures in secondary lymphoid organs, where affinity maturation of B cells takes place following an infection. While GCs are responsible for protective antibody responses, dysregulated GC reactions are associated with autoimmune disease and B cell lymphoma. Typically, ‘normal’ GCs persist for a limited period of time and eventually undergo shutdown. In this review, we focus on an important but unanswered question – what causes the natural termination of the GC reaction? In murine experiments, lack of antigen, absence or constitutive T cell help leads to premature termination of the GC reaction. Consequently, our present understanding is limited to the idea that GCs are terminated due to a decrease in antigen access or changes in the nature of T cell help. However, there is no direct evidence on which biological signals are primarily responsible for natural termination of GCs and a mechanistic understanding is clearly lacking. We discuss the present understanding of the GC shutdown, from factors impacting GC dynamics to changes in cellular interactions/dynamics during the GC lifetime. We also address potential missing links and remaining questions in GC biology, to facilitate further studies to promote a better understanding of GC shutdown in infection and immune dysregulation.

## Introduction

Germinal Centres (GCs) are specialized structures within the secondary lymphoid organs essential for the humoral immune response that form after an infection. B cells in the GCs evolve towards a foreign antigen to progressively improve their affinities (1), by a process referred to as affinity maturation. Affinity maturation is the outcome of somatic hypermutation (SHM) in the genes encoding B cell receptors (BCRs) (2), followed by a selection process aided by follicular dendritic cells (FDCs) and T follicular helper (Tfh) cells within the GCs (3, 4).

**Figure 1:**
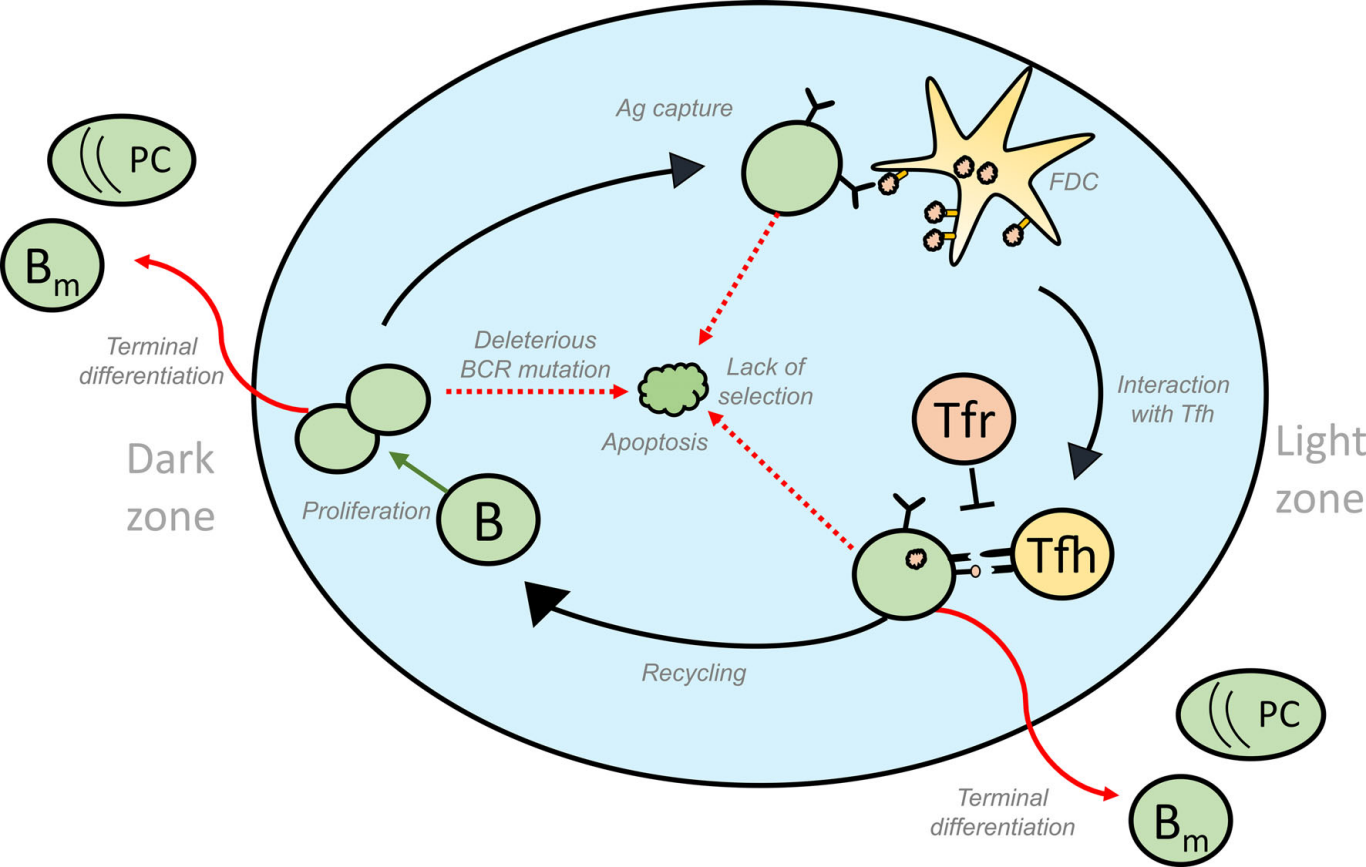
Schematic representation of the different fates of GC B cells that lead to GC volume changes: GC B cells proliferate in the DZ and increase in number. SHM accompanying proliferation might induce deleterious mutations in the BCR gene of some of the B cells, thus activating apoptosis in these GC B cells. Lack of acquisition of antigen, survival signals from FDCs and signals from Tfh cells lead to the apoptosis of GC B cells in the LZ. On the other hand, successful acquisition of these signals could result in the differentiation of the GC B cells into effector cell types such as memory and plasma cells that exit the GC. Alternatively, selected cells can move back to the DZ by a process termed recycling and undergo further rounds of divisions thus contributing to an increase in number of GC B cells. Green and red arrows represent processes that increase or decrease the GC volume, respectively and influence GC shutdown. GC, Germinal Centres; BCR, B cell receptor; FDC, Follicular Dendritic Cells, Tfh, T follicular helper cells, B, GC B cells; Tfr, T follicular regulatory cells; PC, Plasma cell; B_m_ , Memory B cell; Ag, Antigen.

A mature GC consists of two distinct compartments: A dark zone (DZ) and a light zone (LZ) (5, 6). The DZ is predominantly filled with actively dividing B cells called centroblasts, mutating with a high rate resulting in BCR affinity changes (2). A network of FDCs and Tfh cells are present in the GC LZ where the selection takes place. Non-dividing B cells (centrocytes) with mutated BCRs are selected in the GC LZ based on the ability to acquire antigen from FDCs and presenting the processed antigen to the Tfh cells that provide survival and proliferative signals (7, 8). Selected centrocytes can either progress to plasma or memory B cells and egress from the GC, or recycle back to the GC DZ and proliferate (9–11). Iterative rounds of such mutation and selection as GC B cells migrate between the two zones (7), result in a stepwise optimization of B cell affinities towards the locally presented antigen, giving rise to affinity-matured memory and plasma cells (**Figure 1**).

GCs are highly dynamic structures and the evolution of GC arises as a combination of processes at the cellular level: inflow of new founder B cells (12–14), B cell proliferation, apoptosis, and differentiation to effector cell types. Entry of new founder cells and proliferation scales up the number of GC B cells, while apoptosis and differentiation that leads to GC exit counteracts this effect (11). Their contribution to the GC B cell population is dynamic over time as they are influenced by interactions with FDCs and Tfh cells (15), immune complexes trapped on FDCs (16), soluble signals secreted by Tfh cells (17), presence of T follicular regulatory (Tfr) cells (18) and soluble antibodies from plasma cells (19).

GCs show a typical kinetics with an initial phase of growth followed by a contraction phase. In the initial phase, inflow and clonal expansion predominantly increases the number of GC B cells and the GC expands (20) until it reaches a peak size. During the contraction phase, apoptosis and exit exceed proliferation and the GC begins to shrink (11). This already suggests different phenomenological ways in which GC shutdown can occur (schematically shown in the **Figure 1**). Hence, shutdown can be brought about by a decrease in recycling or proliferation, or an increase in apoptosis or terminal differentiation over time. Theoretically, in the absence of an explicit signal, shutdown can be influenced by parameters that control GC events like proliferation, differentiation and apoptosis (11). An impact of DZ to LZ phenotype differentiation speed on the GC lifetime has also been predicted (11) and determines whether the GC reaction will terminate or continue growing in the absence of other shutdown signals (21). The GC response in a lymphoid organ lasts for approximately 3 weeks (22, 23) when induced by model antigens but persist longer in response to viral infections (24). However, lifespan of single GCs has not been determined so far. Tracking the same GC over time *in vivo* is challenging (25), and single measurements might reflect shifted dynamics and are subject to substantial stochastic variation. This hinders the study of single GC dynamics and hence, the experimental analysis of GC shutdown. Consequently, the mechanistic details of natural GC shutdown and which signals induce the shift from expansion to contraction phase are mainly unknown.

**Figure 2:**
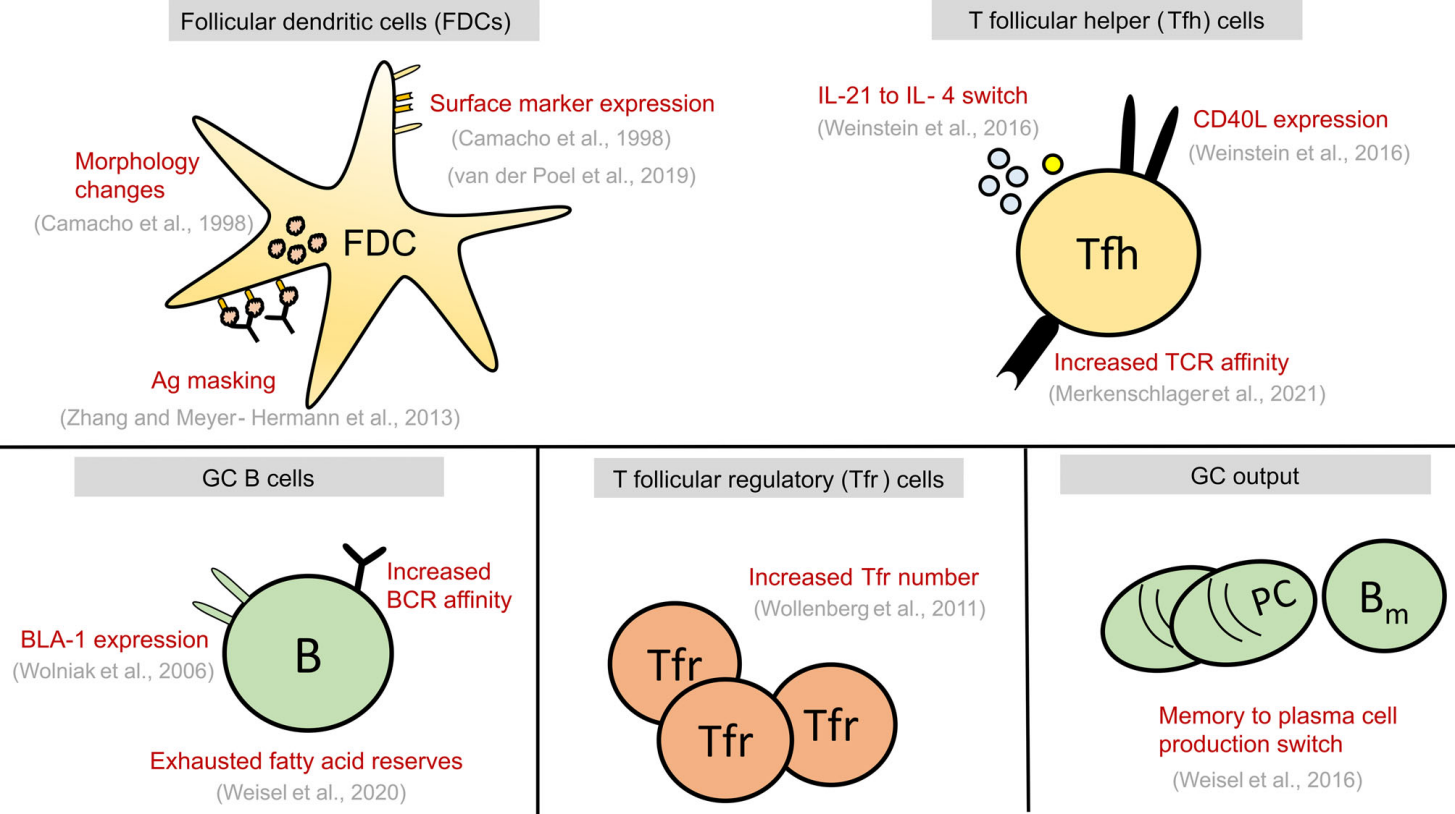
Summary of alterations seen in GC cell types at late stages of GC reaction. These alterations might play a causal role in GC shutdown by influencing the fate decisions of GC B cells shown in **Figure 1** or act as a contributing factor to GC shutdown. GC, Germinal centre; FDCs, Follicular dendritic cells; Tfr, T follicular regulatory cells; Tfh, T follicular helper cells, B, GC B cells; TCR, T cell receptor; BCR, B cell receptor; PC, Plasma cell; B_m_ , Memory B cell; Ag, Antigen.

In this review, we first briefly discuss the various ways by which B cell fates are regulated in a normal GC. Then, we discuss our present understanding of the biological changes (**Figure 2**) that could act as a termination signal and promote GC shutdown by inducing changes in the fates of GC B cells. Finally, we also discuss what we can learn from GCs with extended maintenance phase such as chronic GCs induced by viral infections or dysregulated GCs associated with B cell lymphomas (BCLs).

## Regulation of B Cell Fates in the GCs

As GC kinetics is determined by the balance of apoptosis, proliferation, and differentiation, studies exploring the dynamics of these processes in a normal GC reaction constitute a first approach for a mechanistic understanding of GC shutdown. GC B cell fates are primarily regulated in response to antigen acquisition, soluble cytokines and CD40L signals from Tfh cells. Defects in various B cell intrinsic and extrinsic factors affect normal maintenance and promote GC shutdown (see **Table 1**). However, there is an intense debate over which biological signals are mainly responsible for the normal GC shutdown. The actual cause might vary depending on the type of GC response such as GCs associated with acute or chronic infection, auto-immunity, tumor or allergy. Here, based on the existing knowledge of GC reactions and examples from **Table 1**, we discuss the potential mechanisms that might promote a natural GC shutdown (summarized in **Figure 2**). At first, the different fates of GC B cells relevant to GC shutdown are discussed.

**Table 1:** Summary of GC alterations resulting in premature termination of GCs and the proposed mechanism of action.

Alterations	Observation/proposed mechanism of action	Reference
Activating mutation of CARD11 in GC B cells	Rapid terminal differentiation of B cells	(26)
Inhibition of c-Myc	Prevent DZ re-entry	(27)
c-Rel deletion in GC B cells	Failure in metabolic program directing cell growth	(28)
Bam 32 deficiency in GC B cells	Reduced Tfh recruitment and increased GC B apoptosis	(29)
FDC ablation/absence of FDC	Increased GC B cell apoptosis	(30, 31)
BAFF deficiency	Defect in FDC development and immune complex trapping	(32)
BAFF-R deficiency	Defect in B cell proliferation	(32)
Cr2 deficient mice	Reduced long-term antigen retention	(16)
Absence of T cells	Apoptosis and loss of proliferating cells	(33)
Absence of IL-21 or IL-21 receptor	Reduced GC B cell proliferation	(34)
Absence of Tfh αv integrins	Defect in Tfh accumulation at late stages	(35)
Absence of PD-1	Increased GC B cell apoptosis and reduced cytokine production from Tfh	(36)
anti-CD40L administration	Increased GC B cell apoptosis	(37)
Constitutive CD40 signaling	Early terminal differentiation	(38)

DZ, dark zone; Tfh, T follicular helper cells; GC, Germinal Centres; FDC, Follicular Dendritic Cells; BAFF, B cell activating factor.

### GC B Cell Apoptosis

Extensive apoptosis takes place in both the DZ and LZ compartments of GCs (39). GC B cells spontaneously undergo apoptosis even in the absence of any death signals due to rapidly decaying levels of cFLIP, an inhibitor of apoptosis (40). In addition, FAS activation in B cells leads to apoptosis *via* the activation of caspases (41). Activation of the Fas-mediated pathways can be prevented by interactions with FDCs or acquisition of CD40 signals due to sustained expression of cFLIP (40). Due to the highly active apoptotic program, the default state of the LZ GC B cells is becoming apoptotic, unless they are positively selected by antigen acquisition and Tfh signals (8, 42).

While GC B cell apoptosis mainly happens due to the absence of survival signals and lack of selection in the LZ, DZ B cells undergo apoptosis primarily due to the deleterious mutations induced in the BCR genes during SHM (39). Stewart et al., found that most B cells with deleterious mutations undergo apoptosis in the DZ and are unable to reach the LZ for selection (43). In addition to Fas, various other molecular players such as BCL-2 have been identified to control B cell apoptosis and are believed to act through distinct mechanisms (44). BCL-2 expression can decrease apoptosis of GC B cells by 10-fold (45) but is insufficient to prevent apoptosis due to deleterious mutations induced in BCR genes (43). Jumaa et al. discovered a mechanism by which BCR expression regulates the metabolism of B cells in a BCR signaling independent manner (46). According to this mechanism, BCR regulates changes in endoplasmic reticulum that in turn controls mitochondrial activity. Thus, the loss of BCR expression leads to altered B cell metabolism (46). Consequently, a similar effect might be expected in GC B cells that cannot express functional BCRs due to deleterious mutations.

Apoptosis is considered an important process that is counteracting the extensive proliferation to maintain the constant size of the GCs during their maintenance phase and in Peyer’s patches (PPs) (39). Consequently, changes in apoptosis are associated with changes in GC size (**Table 1**). Deficiency in AID (Activation-induced cytidine deaminase), the enzyme responsible for SHM, decreases the loss of B cells due to damaged BCRs and, thus, increases the size of GCs (47). Complete lack of survival signals such as the absence of FDCs (30) or Tfh cells (33) leads to apoptosis of GC B cells and results in GC termination. Aberrations in Fas and BCL-2 are also associated with B-cell lymphoma (48, 49).

### GC B Cell Proliferation

A characteristic property of GC B cells is the ability to undergo extensive proliferation. GC founder cells divide (6, 22, 25) and increase the GC volume at initial stages. GC B cells positively selected by the Tfh cells recycle back to the DZ (7) to undergo further rounds of division. The speed of cell cycle progression of selected B cells is regulated by T cell help which shortens the S phase (50). The extent of cell division is found to depend on the amount of antigen captured and presented by the B cells to the Tfh cells (51–53). More specifically, the number of divisions of a selected B cell is determined by the level of c-Myc induced by signals from the Tfh cells (54). Cyclin D3 is important for sustaining proliferative expansion of GC B cells (55–57). Pae et al., discovered that Cyclin D3 controls cell divisions in a dose dependent manner, following transient induction of c-Myc by Tfh signals (58).

Changes in the activity of mTORC1 (59) and in expression of FoxO1 (60, 61) are also involved in the regulation of cell division progression in GC B cells. mTORC1 does not dictate the number of cell divisions but is involved in the cell growth prior to clonal expansion, without which the cell cannot divide (59). Proliferation sustains the number of GC B cells, as GCs collapse rapidly due to defects in proliferation or recycling such as c-Myc ablation (27, 62) or c-Rel deficiency (28).

### GC B Cell Terminal Differentiation

Signals from Tfh cells induce the differentiation of certain GC B cells into precursors of output cells, i.e., plasma or memory. These differentiated cells typically exit the GC and decrease the GC volume. It is widely believed that the nature and intensity of Tfh cell signals determine the fate of selected cells although a clear understanding is lacking. Wang et al, discovered that development of memory B cells is dependent on IL-9 produced by Tfh cells (63). Ise et al, discovered a population of LZ B cells that is prone to become plasma cells due to stable interactions with Tfh cells suggesting that differentiation to plasma cells is dependent on the GC B cell-Tfh cell interaction strength (64). However, Kräutler et al. proposed a two-signal based mechanism in which differentiation is initiated by signals delivered to B cells during antigen engagement and Tfh cell signals are only required to complete the process (65). It is also not understood whether the B cells directly differentiate and exit the GC after Tfh selection or recycle and divide further in the DZ before final differentiation and GC exit (52). The latter theory was supported by Radtke and Bannard, who showed that dividing GC B cells in the DZ express low levels of BLIMP-1 and are more sensitized to plasma cell differentiation (66). A faster terminal differentiation can promote faster exit of B cells from GC and leads to early GC termination (38). On the other hand, lack of differentiation and GC exit can lead to excess accumulation of B cells in the GCs (67). In general, it has been accepted that early waves of low affinity cells exiting the GC are enriched in memory cells, while later high affinity cells generate more plasma cells (68–70), suggesting a gradual switch between the decision of memory to plasma cell differentiation (71). CD20 is involved in maintenance of the resting state of Ramos B cell by regulating receptor organization on the surface of the cell and the loss of CD20 induced the differentiation of naïve B cells into plasma cells (72). Similarly, whether changes in the expression or ligation of CD20 is involved in the GC B cell differentiation into plasma cell needs to be investigated. Further work clarifying the signals promoting differentiation or recycling and their dynamics during the GC reaction would help improving knowledge on GC volume changes due to these processes.

## Morphological and Functional Changes of Follicular Dendritic Cells

Follicular dendritic cells (FDCs) are necessary for the maintenance of GC reactions as disrupting the FDC network results in premature termination of GCs (30). Similarly, GCs formed in the absence of FDCs are not maintained at later stages (31). FDC disruption results in unavailability of immune complexes carrying the target antigens and lack of cytokines or survival factors such as BAFF (B cell activating factor). Absence of BAFF and BAFF receptor (32) or defect in long term retention of antigen due to the lack of complement receptors (16) result in unsustained GC responses.

FDCs undergo progressive development during the GC response which is observed as changes in morphology and expression of surface markers such as CD23 (6), ICAM-1 (73), VCAM-1 (74) and FcγRIIB receptors (74, 75). Environmental signals have been shown to influence the functional ability of FDCs (76). Cytokines such as IL-2 and IL-10, could also alter the morphology of FDCs by influencing their contractility (77).

Intrinsic defects in aged stromal cells are associated with changes in magnitude and output of GC responses (78). Similarly, such changes in the nature and differentiation state of FDCs over time potentially modulate the interaction of B cells with FDCs (6), but it is unknown if similar changes occurring at late stages trigger GC shutdown. As FDC maintenance requires lymphotoxin signaling delivered by the B cells (79, 80), changes in the differentiation state of FDCs by continuous interactions with GC B cells may promote GC shut down (81) by altering survival signals and/or antigen availability.

Keşmir and De Boer have predicted that limiting antigen rather than Tfh cell help drives the shutdown of GCs as only a few T cells are sufficient to sustain GC reactions (82). A decrease in antigen availability over time would reduce the uptake of antigen by GC B cells and in turn decrease the intensity of Tfh cell signals received. This can terminate the GCs by increasing the fraction of B cells undergoing apoptosis and/or by decreasing the Tfh cell induced proliferation of B cells. As a large amount of antigen is observed in follicles for an extended period of time (83), it was believed that GC shutdown is unlikely to be due to a decrease in antigen availability. However, the antigen access of GC B cells could vary due to the above-described changes in the FDC morphology. A complex organization and non-uniform distribution of immune complexes on the FDC surface has been identified (83, 84). Alterations in FDC morphology leading to a loss of iccosome generation and burial of antigen in the form of pockets could be the reason behind termination of GCs (85). Investigating the organization of immune complexes and receptors on the FDC surface at different stages of the GC reaction might unravel the role of FDCs in GC shutdown.

Heesters et al. found that immune complexes in the FDCs undergo periodic cycling (86, 87), suggesting a mechanism for antigen retention (83, 88, 89). As changes in antigen presentation dynamics might also terminate GCs, it can be speculated that GC termination might be due to modulation of antigen cycling dynamics during the GC reaction. Although changes in the morphology and functional state of FDCs might induce changes in antigen presentation dynamics, there is no experimental evidence that antigen cycling is dynamically modulated in the GC FDCs.

Alternatively, phenotypic changes in FDCs could positively influence GC B cell differentiation rather than limiting antigen access. This mechanism could also potentially terminate GCs by promoting differentiation and exit of GC B cells (81).

## Feedback Regulation by Soluble Antibodies

Negative feedback due to soluble antibodies is another mechanism that could decrease the antigen access of GC B cells. Soluble antibodies can suppress or enhance the antibody response, when passively administered with an immunogenic antigen (90). Similarly, maternally derived antibodies can suppress the vaccination response of offspring by influencing the output of GC reactions which can be partially overcome by increasing the dose of antigen (91). Suppression of B cell responses by soluble antibodies can occur due to epitope masking of the administered antigen (19, 92–94).

Further, exogenous antibodies administered are able to enter the GCs, are found deposited on the FDC network and alter GC kinetics and affinity maturation (19). Consequently, soluble antibodies produced from plasma cells might mask the antigen on FDCs and limit available antigen (21, 95). Accordingly, one theory is that the endogenous soluble antibodies terminate the GC by decreasing antigen access over time (19). Mathematical modeling also predicted that high concentration of antibodies can shut down GCs (19, 96). The existence of epitope-specific antibody feedback could shift the B cell response away from immunodominant epitopes by enhancing B cells recognizing other epitopes (93, 97), and generate complex GC dynamics. In the context of secondary stimulation, pre-existing high affinity memory B cells might induce a premature end to the GC reaction due to high antibody feedback derived from differentiation of memory B cells to antibody-forming plasma cells (97). This theory is supported by the finding that reentry of reactivated memory B cells into GCs is limited (98).

It is hard to test the mechanism by which the soluble antibodies influence GC shutdown because lack of soluble antibodies will not only disrupt a potential antigen masking phenomenon but also decrease the clearance of antigen. Both the lack of soluble antibodies (19) and long term persistence of immune complexes (99) are associated with long-lived GCs. However, Bergström et al. showed that the inhibitory effect of passively administered IgG does not correlate with the clearance of antigen from the lymphoid organ and further, inhibition of antibody response was seen in FcRγ knockout mice which lacks the clearance mechanism by antibodies suggesting that suppression is most likely due to antigen masking (92).

On the contrary, early waves of antibodies could support the GC reaction by facilitating the transport of antigen to FDCs in the follicles. Although small antigens can directly enter B cell follicles through conduits, larger antigens are transported by B cells in a complement-dependent manner (100–103) which relies on the formation of immune complexes. This enhancing role of antibodies is likely restricted to early stages of the GC reaction. Whether this mechanism contributes to the maintenance of chronic GCs with persistent antigen due to replicating pathogens needs to be examined.

GCs might also communicate by the exchange of soluble antibodies (19). As newly produced or injected antibodies quickly distribute over the organism, it is likely that antibodies produced by one GC will appear in other GCs. But it is unknown whether the GC shutdown is extrinsically regulated by other GCs.

## Progressive Changes in T Follicular Helper Cells

Tfh cells are required to maintain the GC reactions (33). Like FDCs, dynamic changes are also observed in Tfh cells during the course of a GC reaction. Tfh cells switch from a primarily IL-21 to an IL-4 producing state (104). Changes in surface ligand expression such as CD40L are also observed as the GC evolves (104).

Affinity of TCRs could influence the GC lifetime (105) by impacting the GC B cell fate decision. Hence, the repertoire and specificity of Tfh cells might be critical in determining the GC longevity. Recent findings suggested that the Tfh cells undergo selection similar to GC B cells (106). In this process, Tfh cells with high affinities towards the pMHC presented by B cells are selected (106). Implications of such Tfh selection on GC shutdown are presently unknown.

Lack of IL-21 signaling (34) or block of CD40L (37) also result in premature termination of GCs as recycling and proliferation of GC B cells cannot be sustained. On the other hand, constitutive expression of CD40L on B cells mimicking excessive CD40 signaling also resulted in earlier termination of the GCs (38, 107). Understanding this bimodal effect of Tfh cell signals might help interpret the role of Tfh cells in maintenance vs shutdown.

A positive feedback loop of ICOS-ICOSL signaling promotes brief interactions between B and Tfh cells with enhanced surface engagement (108). Pratama et al., have shown that miRNA-146a regulates ICOS-ICOSL signaling in GCs and loss of this miRNA results in excess numbers of Tfh and GC B cells due to increased ICOSL expression on GC B cells (109). As miRNA-146a expression peaks at late stages of GC responses when the Tfh cell response declines (109), this suggests that reduced ICOS-ICOSL signaling might influence the GC dynamics at late stages leading to GC termination.

Absence of PD-1 on Tfh cells also resulted in smaller GCs at late time points due to increased GC B cell apoptosis (36). In summary, these findings suggest that GC shutdown can be altered by changes in Tfh cell signals. However, examining the role of Tfh cells in shutdown faces numerous challenges. B-T interactions in GCs are rather transient (110) compared to B-T interactions at the T-B border zone (111), making it hard to monitor them. As T cell help received by GC B cells is dependent on presented antigen (7), it is possible that underlying changes during shutdown are primarily arising from changes in antigen uptake by B cells as described in previous sections. Moreover, Tfh and GC B cells are known to maintain and influence each other in murine GCs (106, 112). Such intricate mutual dependence makes it hard to distinguish the cause and the effect (105).

Another mode of Tfh cell regulation is inhibition by antigen presentation of plasma cells (113), warranting further studies on maintenance of Tfh cells and the interplay with B cell.

Tfh cells can also migrate between GCs (114), contributing to intercommunication between different GCs. It remains speculative whether GCs regulate the dynamics and shutdown of neighboring GCs by the exchange of Tfh cells.

## Suppressive Nature of T Follicular Regulatory Cells

Immune suppressive mechanisms of regulatory T cells (Tregs) play a role in controlling the magnitude of immune responses (115). T follicular regulatory (Tfr) cells, expressing CXCR5, PD-1 and Foxp3 were identified as Treg subset found in GCs (18, 116, 117). Dynamics of Tfr cells suggest that they might play a role in the GC termination. Tfr cells increase in number at the peak of the GC reaction and the Tfh/Tfr ratio decreases during the contraction phase (18, 118).

Studies on the influence of Tfr cell deletion on GC kinetics or GC size reported contradictory results and different subsets of Tfr cells might have different effects (119). These contradictory results could also partly be explained due to differences in experimental approaches and markers used to deplete Tfr cells. Even though CXCR5 is generally targeted to deplete Tfr cells in GCs, it was shown that CXCR5 negative Tfr cells can still access the GCs (120), suggesting a need to better characterize the markers and behavior of Tfr cells in order to specifically deplete them.

However, characterization of Tfr cells remains challenging. For instance, though Tfr and Tfh cells share phenotypic characteristics and are yet distinct (117), unique suppressive T cell subsets which show moderate characteristics of both cell types were found in human tonsils (121). Similarly, in aged mice, IL-10 producing Tfh cells accumulate which is associated with impaired responses (122).

Indeed, in murine Peyer’s patches, Foxp3+ T cells can transform into Foxp3- Tfh cells, showing that Foxp3 expression is plastic (123). Inducing Foxp3 expression is sufficient to convert Tfh to Tfr like cells (124), suggesting that they might cross-differentiate and influence the magnitude of Tfh cell signals over time. Mathematical analysis of the dynamics of naïve T cells and Tregs predicted the contribution of the conversion to pTregs towards maintaining T cell homeostasis and suggested it could be more prominent in lymph nodes than in the spleen (125). Hence, organ specific differences in trans-differentiation of Tfh to Tfr cells and vice versa might be expected and require further investigation.

Several mechanisms have been proposed to explain the influence of Tfr cells on the GC response, which includes a direct inhibitory action on B cells and indirect effects by suppressing Tfh cells (117, 119, 126, 127). Wing et al. and Sage et al. showed that Tfr cells exert their inhibitory action *via* the inhibitory receptor CTLA-4 (126, 128). In general, CTLA-4 inhibits T cells by outcompeting the CD28 co-stimulatory receptor by binding to B7 ligands (129) on antigen-presenting cells and/or by the trans endocytosis of B7 ligands (130, 131). However, it is not clear whether the inhibitory action of CTLA-4 on GCs depends on trans-endocytosis as the expression levels of B7 ligands on GC B cells in the absence of CTLA-4 (126, 128) showed contradictory results. The role of Tfr cells in GC termination will become easier to probe as future studies improve our understanding of their mechanism of action and influence on GCs.

## Potential Role of Tingible Body Macrophages

Phagocytic macrophages called tingible body macrophages (TBMs) present in GCs (132) might play a role in downregulating the GC reaction. Smith et al. observed that TBMs are capable of suppressing IL-2 induction in T cells upon stimulation by B cells in an *in vitro* culture system through a prostaglandin dependent mechanism (133).

Kinetics of TBMs follow the GC reaction kinetics, and it has been suggested that they play a role in regulating the magnitude of GC reactions (134). However, it is not known whether TBMs modulate the GC dynamics or vice versa. TBMs are capable of endocytosing iccosomes from FDCs (135). Thus, there is a potential inhibitory effect of TBMs on GC by limiting antigen availability which requires further investigations.

Impaired apoptotic cell clearance in the GCs by Mer-deficient TBMs increases the GC B cells at different stages of the GC response (136). Moreover, impaired apoptotic clearance is associated with autoimmune diseases such as systemic lupus erythematosus (137, 138). Even though this rarely studied cell type might not be sufficient for the shutdown, a modulatory effect on GC shutdown could be expected.

## Role of Innate Signals

Adjuvants used to enhance the magnitude and longevity of antibody responses have also been shown to alter the kinetics of GC reactions (139). Adjuvants are capable of influencing the differentiation of Tfr (140) and Tfh cells (141) and could alter the Tfh : Tfr ratio even in GC responses towards the same antigen (140). The mechanisms of action of different adjuvants are still being elucidated, but many adjuvants act as ligands for innate immune receptors such as Toll-like receptors (TLRs) (142).

Several studies have demonstrated that TLRs modulate the magnitude and quality of GC responses but it is not known whether only the early development of GCs or also late stages of the GC reaction are affected (143). TLRs enhance the GC response by acting on dendritic cells and/or B cells (143). For instance, TLR9 impacted the GC reaction by acting on both dendritic cells and B cells (144). Action of TLR9 on dendritic cells enhanced the number of Tfh and GC B cells, while TLR9 signaling in B cells was associated with increased ICOS on Tfh cells and a reduced number of Tfr cells (144). FDCs express TLR4, which is upregulated during GC development and loss of TLR4 signaling resulted in a decreased size of GCs (73). Antigen deposition on FDCs has been shown to be enhanced by the TLR ligand-based adjuvant PorB (145).

Kasturi et al. have shown that a combination of TLR4 and 7 ligands can synergistically enhance the antibody response and the persistence of GCs when compared to administering either TLR ligand with antigen (146), suggesting that TLRs can be targeted to modulate the lifetime of GCs. Although TLRs can impact the GC longevity, their role in GC shutdown has not been investigated so far, and would be of importance to understand the relative contribution of different adjuvant strategies to vaccine success.

## Metabolic Inhibition

The metabolism of GC B cells has been given minor focus so far, due to technical difficulties in analyzing the GC micro-environment (147, 148). It was acknowledged that GCs are a nutrient-poor and hypoxic environment especially in the LZ (149), possibly required for proper selection of B cells. Weisel et al., found that GC B cells exhibit a rather low glucose uptake and instead use fatty acids as a major source of energy (150). However, subsets of GC B cells such as the positively selected cells might transiently use a higher glycolytic program (150), as mTORC1 activation is associated with increased glucose uptake after DEC205-OVA induced GC B cell interaction with Tfh cells (59). Blockade of glutaminolysis pathways with the DON glutamine analogue leads to GC shrinkage and could be mediated by the Tfh cell sensitivity to glutamine (151). Interestingly, several transcription factors controlling proliferation of centroblasts are also metabolic sensors, such as c-Myc, mTOR, FoxO, suggesting that metabolism can regulate GC dynamics. For instance, mTORC1 controls the anabolic program by regulating the synthesis of lipids, glycolytic flux and by promoting protein synthesis (152), ultimately supporting B cell proliferation. The RNA binding protein PTBP1 that is highly expressed in the positively selected GC B cells, regulates alternative splicing of genes including *Pkm* (M-type pyruvate kinase), *Tyms* (Thymidylate synthase), suggesting a post transcriptional control of glycolytic flux and nucleotide synthesis (153).

Metabolic changes have been observed in the GC B cells as the GC matures. Though GC B cells are able to oxidize both endogenous and exogenous fatty acids, they rely more on exogenous fatty acids as the GC progresses, which might be obtained *in vivo* from dying B cells (150). This is presumably because of an exhausted endogenous supply of fatty acids (150). The significance of such metabolic changes in GC shutdown is unexplored.

In *Plasmodium* infection, short-lived plasmablasts have been suggested to inhibit GC function by imposing glutamine deprivation (154). This finding suggests that glutamine competition due to the plasma cells in extrafollicular areas might contribute to GC shutdown. As malaria is associated with decreased serum levels of glutamine and anemia, this needs to be shown under physiological metabolic balance in a normal infection.

Son et al., showed that inhibiting the Endoplasmic reticulum associated enzyme, stearoyl-CoA desaturase suppresses Tfh and GC B cell responses (155). Inhibiting this enzyme impaired Tfh cell maintenance and disturbed the balance between Tfh and Tfr cells by enhancing Tfh apoptosis (155). This suggests an impact of metabolic changes on GC responses, but whether such metabolic changes could be responsible for GC shutdown and how such metabolic changes might arise remains unclear.

## Dynamic Changes in GC B Cell Characteristics

Studies have revealed characteristic changes in the GC B cells at late stages of GC responses, in addition to the metabolic changes. Very late stage GC B cells were resistant to the antigen depletion (156), suggesting that the maintenance of these GC B cells could be different from the early stages. In addition, a fraction of GC B cells expressing the BLA-1 marker was found to decrease at late stages (157), but its functional significance is unknown. Further, there is a shift in the production of memory cells to plasma cells during the course of the GC reaction (71).

These findings suggest that we do not fully understand the behavior of late GC B cells and there could be undiscovered negative feedback mechanisms acting at late stages which might also play a role in the termination of the GC response. Such mechanisms could be unveiled by studies focused on late stages of GC responses. Similarly, computational modeling suggested that a hypothetical proliferation signal that decays over time would result in reduced number of divisions as GC progresses and leads to GC termination (81). Extensive monitoring of time-dependent changes in GC B cells in parallel with other GC cell types can reveal a great deal of information.

## Impact of B Cell Repertoire and GC Seeder Cell Composition

Composition of B cell repertoire is an important determinant of GC B cell recruitment (158). Mathematical modeling has predicted that the affinity of seeder cells influences the efficiency of GC reaction (159). As low affinity and frequency of antigen specific cells in the repertoire can lead to poor vaccination responses, it has been suggested to evaluate these parameters during vaccine development (160). Lifetime of GCs might also be influenced by the B cell repertoire and the founder cell composition of GCs.

In response to a secondary immunization, only limited participation of memory B cells has been observed in recall GCs (98). This finding can be explained by antibody feedback arising due to the differentiation of memory B cells into plasma cells, as the soluble antibodies produced would prevent competitive participation of memory B cells in the GCs (97). Accordingly, recall GCs allow for affinity maturation to a new epitope (97) unlike the concept of “original antigenic sin” (161) and also suggests a role of antibody feedback in the regulation of GC lifetime. As certain subsets of memory cells are capable of participating in GC responses (162), determining factors governing the composition of seeder B cells in primary and recall GC responses might allow for a better understanding of the regulation of GC lifetime by founder cells.

Apart from the composition of seeder cells, activation of naïve B cells and influx of new founder cells can continue after the GC is established (12, 13) and might extend the longevity of ongoing GC responses. Such continued influx of new founder cells might likely contribute to persistence of GCs seen in Peyer’s patches and viral infections.

## Hints From GCs With Diverse Kinetics

Perturbation experiments have revealed that defects in a large number of factors can trigger GC shutdown (**Table 1**). Although such perturbations help infer the role of different factors in maintaining or terminating the GC, they only provide a limited understanding of the primary mechanism of GC shutdown. Studies have shown that the lifetime of GCs varies depending on the nature of the antigen stimuli (24, 163) and other factors such as the organ under consideration (164).

Li et al. compared the microbial exposure of germ-free mice at systemic or intestinal mucosa, and found differences in the diversity of the resulting BCR repertoire (165). It would be interesting to test the differences in GC dynamics and shutdown with different routes and sequences of microbial administration. Individual GCs in a lymph node have widely varying rate of loss in clonal diversity (166). Although the exact mechanism behind such differences in clonal evolution of individual GCs was not addressed so far, the observed heterogeneity despite the dynamic exchange of Tfh (114) and potential intercommunication due to soluble antibodies (19), raises the question whether individual GCs have the same lifetime and shutdown mechanisms and whether differences in the pace of clonal evolution are associated with differences in GC population kinetics, maintenance, and shutdown. It may be envisioned that the relative affinity of B cells in a single GC could regulate its dynamics. Although it is unknown whether the mechanism of shutdown is conserved under diverse immunization conditions, comparing the differences in GC response to such diverse conditions including natural viral infections, constitutive GCs seen in Peyer’s patches (**Table 2**) can provide some hints about the physiological signals that limit the GC lifetime.

**Table 2:** Summary of characteristic differences seen in the GCs induced by chronic viral infections, GCs of Peyer’s patches and GC-derived B cell lymphomas when compared to the transient GC responses induced by model protein antigens.

Condition	Characteristics	References
Chronic viral infections	Persistent GCs and efficient affinity maturation	(167)
	Alterations in Tfh and Tfr proportions	(168, 169)
Peyer’s patches	Sustained maintenance phase	(39)
	Chronic antigen stimulation	(170, 171)
	Rapid clonal turnover	(172)
	Il-4 and Il-12 expressing Tfr cells	(173)
	High IL-21 expression in Tfh cells	(174)
	IgA as predominant antibody isotype	(175)
	FDCs producing high levels of CXCL13, BAFF and TGF-β1	(76)
B cell lymphomas	Disruption of GC B cell apoptosis. Example: BCL-2 translocation	(176)
	Increased B cell divisions. Example: overexpression of c-Myc	(177)
	Block in the terminal differentiation. Example: Activated B cell like – Diffuse Large B cell lymphomas	(67, 178)
	Preferential re-entry of cells into GCs due to BCL-2 translocation	(179)
	Altered intrinsic apoptotic pathways. Example: EBV infection	(180)

Tfh, T follicular helper cell; Tfr, T follicular regulatory cell; FDC, Follicular dendritic cell; EBV, Epstein-Barr Virus; BAFF, B cell activating factor; TGF, Transforming growth factor.

## Chronic GCs Due to Viral Infections

Chronic viral infections are capable of inducing long-lived and persistent GCs (181). For instance, after infection with VSV (Vesicular stomatitis virus), GCs were detectable up to 100 days after immunization (24). Chronic viral infections are associated with efficient affinity maturation and production of memory and plasma cells due to persistent GC responses (167). Persistence of GCs and the long-term maintenance of antibody titers is believed to be associated with persistent antigen but there is no direct evidence for this. Also, the resolution of the infection does not always result in GC termination. In the case of influenza virus infection, GCs are formed when the infection is almost resolved but the GCs are still long-lived (182, 183). Sustained GC responses could be due to the persistence of residual viral antigens (184) even after the resolution of infection. In addition to antigen persistence, there are also changes in Tfh and Tfr cell proportions in some viral infections (168, 169). However, the primary reason for the observed longevity of GCs has not been addressed so far.

## Constitutive GCs in Peyer’s Patches

Constitutive GC reactions are observed in Peyer’s patches (PPs) of the small intestine, a part of the MALT (Mucosal Associated Lymphoid Tissue). These GCs characteristically have a constant GC volume, which reflects their sustained maintenance phase (39). The key factor that sustains the PP GCs is believed to be the chronic antigen stimulation by intestinal microbiota and antigen encountered in the food (170, 171). In the gut associated GCs, clonal turnover happens at a rapid rate and the rate of selection varies depending on the complexity of microbiota (172). GCs in the PPs also differ from the conventional GCs in lymph nodes and spleen in terms of the Tfh, Tfr cells, FDCs and isotype of antibodies produced (185). Tfr cells in PPs express Il-4 and Il-21 (173) unlike the Tfr cells in conventional GCs. The impact of these differences is not understood. These GCs host a high IL-21 expressing Tfh cell population, which when disrupted reduces the number of GC B cells (174).

In addition to Tfh and Tfr cells, PP FDCs are also considerably different from FDCs in the lymph node in their gene expression profile and produce high levels of CXCL13, BAFF and TGF-β1 due to stimulation by bacterial products and retinoic acid (76). Another major difference is the isotype of antibodies produced; PP GCs predominantly produce IgA antibodies (175) as opposed to IgG in the case of peripheral lymphoid organs. Although, in the latter, class-switch recombination predominantly occurs outside the GCs (186), the isotypic differences of GC B cells and antibodies produced might influence the GC dynamics and is worth investigating.

Microbiota might induce GC formation in a BCR independent manner by the interaction with innate immune receptors (187) and hence, the antigen recognition ability of GC B cells might impact the GC longevity and dynamics. Identifying the key factors promoting the constitutive nature of these GCs would be of importance in enhancing GC response towards vaccination.

## Dysregulation in GC-Derived B Cell Lymphomas

GC B cells have a typical gene expression profile to facilitate affinity maturation. Acquisition of antigen and Tfh cell signals dynamically regulate the gene expression profile of GC B cells and promote their transition through different phases of the GC reaction. Intrinsic defects in the GC B cell gene expression programs that influence apoptosis, proliferation and differentiation potentially disrupt normal GC shutdown and trigger lymphomagenesis [reviewed in (188)]. For instance, translocation of BCL-2 can prevent GC B cell apoptosis and increase the number of GC B cells (176). Overexpression of transcription factors such as c-Myc can promote sustained proliferation of GC B cells (177). Further, a block in the differentiation of the GC B cells is seen in ABC-DLBCLs (Activated B cell like – Diffuse Large B cell lymphomas) which result in an accumulation of B cells within the GCs (67, 178). Such intrinsic B cell defects lead to a premalignant state which might undergo malignant transformation when multiple mutations accumulate over time and is facilitated by SHMs in the GCs. In addition, it has been shown that BCL-2 overexpressing cells might preferentially re-enter GCs upon subsequent antigen challenge and can drive lymphomagenesis due to repeated accumulation of diverse mutations (179). There is also evidence suggesting that intrinsic apoptotic pathways of GC B cells are altered by the EBV (Epstein-Barr Virus), a virus associated with Burkitt’s lymphoma facilitating the survival of EBV infected GC B cells (180). Hence, knowledge of the role of B cell genetic defects not only helps to promote an understanding of how the gene expression program of GC B cells dictate normal shutdown but also their dysregulation in lymphomagenesis which could further help to identify targets to block the progression of lymphomas.

## Implications in Dissolving Ectopic GCs

In addition to the applications of understanding GC shutdown in designing treatment strategies for GC associated B cell lymphomas, it is also important for disruption of ectopic GCs formed in a certain proportion of patients with autoimmune diseases (189, 190). For instance, ectopic GCs are found in the synovium of Rheumatoid Arthritis (RA) (191, 192), salivary glands of sjögren’s syndrome (193, 194), meninges of multiple sclerosis (195) and thymus of myasthenia gravis patients (196).

Significant correlation between the presence of rheumatoid factors and follicles in the synovium of RA patients (197) and the increase in the affinity of auto antibodies (198) over time suggest that their source could be ectopic GCs although it is not proven (199). These primary follicular structures include FDCs, B cells and T cells organized into typical GC-like structures, which, in contrast to their more static counterparts in secondary lymphoid tissues, were predicted as structures dynamically changing in dependence on chemokines (200) and are thought to promote pathogenesis. They can be disrupted by anti-CD20 antibodies which collapse the B cell follicles (192). It has also been shown that CD8 T cells are important in sustaining the activity of such GCs as disruption of CD8 T cells disrupted GCs (201).

Understanding the natural mechanism and regulation of GC shutdown might help identify efficient ways to target such ectopic GCs. However, as these ectopic GCs might differ from normal GCs, factors in their maintenance apart from the conventional shutdown mechanisms must be determined.

## Implications in Vaccine Development

Considering the requirement for effective vaccines against pathogens such as influenza, HIV and more recently, SARS-CoV-2, main focus of several studies is to develop strategies to enhance GC responses by vaccination (202–206). For vaccine development, apart from the choice of antigen and adjuvant, modulation of antigen dynamics has been identified as a strategy to enhance GC responses. Persistent antigen deposition observed during a natural viral infection gave rise to the concept of slow delivery immunization (207). Administering escalating doses of antigen increases immune complex deposition on FDCs and enhances the GC response (204). Similarly, compared to conventional bolus immunization, slow delivery immunization in non-human primates resulted in enhanced neutralizing antibody responses against HIV envelope protein (203). Slow delivery of antigen also enhanced the number of GC B cells, Tfh cells, diversity of GC B cells and antigen retention on FDCs (203).

Longevity of GCs is a key factor that influences the quantity and quality of GC responses. When combined with efficient GC induction, extending the longevity of induced GCs may improve vaccine efficacy. Furthermore, magnitude of recall GC response is limited when compared to the primary GC response (22). Factors influencing the lifetime and magnitude of secondary GCs need to be determined to modulate the GC response to booster immunization. Therefore, a better understanding of the mechanism of GC shutdown in primary and recall GC responses can help identify effective ways to enhance the longevity of GCs and might revolutionize the development of vaccines.

## Challenges in Understanding GC Shutdown

Inferring the mechanistic details of GC shutdown from present evidence poses several challenges. Firstly, alterations in GC processes do not always lead to observable changes in GC kinetics and shutdown. For instance, GC kinetics in CD80 deficient mice (208) remains unaltered despite lowered production of plasma cells, as changes in the production of memory cells and apoptosis take over. Also, while TLR9 signaling enhances the magnitude of GC responses on day 14, there is no difference observed at later stages such as on day 21 (144). Hence, changes that influence GC magnitude must be distinguished from those that influence GC kinetics.

Secondly, the dependence of GC progression and kinetics on various factors vary under different conditions. For example, Bam32 deficient mice exhibit a defect in GC maintenance with low doses of SRBC injection but not at high doses (29). Hence, the variation of antigen amount might help disentangle the different mechanisms in their importance for the shutdown. Similarly, GC maintenance was affected by the complete lack of CD40 but was intact with CD40 haploinsufficiency (64). Dependence of GC shutdown on different factors might also vary with the complexity of antigen used for immunization. GC response to complex antigens with different epitopes and Tfh receptor specificities, show GC B cells that have very low affinity or do not bind to the antigen used (209). Such an ability of non-cognate B cells to participate in GC reaction (210) might hide underexplored reasons for peculiar GC dynamics.

Finally, although several factors could alter GC termination, it is experimentally difficult to test which ones play a role in natural termination of GCs. Further, mechanistic understanding of GC shutdown is challenging due to incomplete understanding of how changes in cellular interactions promote molecular changes in GC B cells and how this in turn influences further cellular interactions.

## Mathematical Models for Understanding GC Shutdown

Mathematical models as a tool for exploring GCs (211) have contributed substantially to GC research by predicting the recycling of GC B cells (9, 10), limiting nature of Tfh help (95) and antibody feedback (19). Wang et al., developed a stochastic GC model, and predicted that quantity and quality of GC responses can be tuned by the efficiency of T-B interactions (212). De Boer and Perelson developed mathematical models considering selection mechanisms based on density or diversity of pMHC presentation by GC B cells to investigate the evolution of broadly neutralizing antibodies (213). Increasing the breadth of Tfh cell repertoire and magnitude of Tfh responses were found to enhance the selection of broadly reactive GC B cell clones and lead to a quick development of broadly neutralizing antibodies (213).

Due to the technical difficulties in testing the different mechanisms experimentally and non-intuitive behavior of GCs, mathematical modeling studies were more prevalent in exploring the mechanism of shutdown. Predictions of mathematical models have suggested various ways of GC shutdown and the parameters critical for explaining the GC dynamics. Identifying potential mechanisms of shutdown by mathematical models and experimentally testing the predictions might efficiently identify the mechanism of GC shutdown.

## Concluding Remarks

Although several mechanisms such as changes in antigen access or nature of Tfh cell signals could promote GC shutdown, identifying the natural cause of GC shutdown remains a challenge. These mechanisms might be inter-related due to complex dependencies in the GCs. A likely response to the GC shutdown mechanism would state that GCs are terminated in a coordinated action of many mechanisms. In this perspective, a partial disruption of one mechanism would not necessarily induce chronic GCs. Nevertheless, several lines of evidence point to antigen availability as a key requirement for GC B cell and Tfh maintenance and suggest a role of antigen limitation. Consequently, a better understanding of antigen availability and accessibility to GC B cells might help solve the long-lasting debate about antigen vs Tfh help as a shutdown signal.

Factors determining the alternative fates of GC B cells are crucial for understanding GC shutdown. Given that loss of function attempts alone might not be informative in the context of GC shutdown, a combination with gain of function mutation studies is necessary. It is important to understand how different factors combined determine the lifetime of GCs and timing of shutdown. *In vitro* GC development and the approach of synthetic biology might also be powerful in gaining a more detailed understanding. A better characterization of progressive changes happening in GCs, in terms of cellular dynamics, interactions, metabolism, surface markers and other features and the basis of such changes is also important. As GCs develop asynchronously and differ from one another, longitudinal imaging of GCs for sufficiently long periods of time is an important requirement for investigation of GC shutdown. Studies of the late phases of GC reactions and monitoring the GC contraction phase can provide more information on the mechanism of GC shutdown and how different factors coordinate to regulation or pathological dysregulation of the GC contraction.

It is also not clear whether the mechanism of GC shutdown and their interplay could be variable under different conditions. Main reason leading to variability in the lifetime of GCs induced under different conditions needs to be elucidated. In the context of multiple asynchronous GCs, mechanism of shutdown of individual GCs rather than the overall response needs to be addressed by future studies. Understanding the mechanism and factors regulating GC shutdown would allow for modulation of GC lifetime to improve vaccination responses. Although the primary focus of the review was to motivate studies on understanding natural GC shutdown, it also highlights a need to better understand the diverse ways in which GC shutdown is dysregulated for potential therapeutic developments.

## Author Contributions

TA, SB, PR, and MM-H wrote the manuscript. All authors contributed to the article and approved the submitted version.

## Funding

TA was supported by the European Union’s Horizon 2020 research and innovation programme under the Marie Sklodowska-Curie grant agreement no. 765158. PR was supported by the Human Frontier Science Program (RGP0033/2015).

## Conflict of Interest

The authors declare that the research was conducted in the absence of any commercial or financial relationships that could be construed as a potential conflict of interest.
